# Forecasting readmission in COVID-19 patients utilizing blood biomarkers and machine learning in the Hospital-at-Home program

**DOI:** 10.3389/fmed.2025.1469245

**Published:** 2025-03-26

**Authors:** Maria Glòria Bonet-Papell, Georgina Company-Se, María Delgado-Capel, Beatriz Díez-Sánchez, Lourdes Mateu-Pruñosa, Roger Paredes-Deirós, Jordi Ara del Rey, Lexa Nescolarde

**Affiliations:** ^1^Department of Hospital at Home, Hospital Universitari Germans Trias i Pujol, Barcelona, Spain; ^2^Department of Medicine, Faculty of Medicine, Universitat Autònoma de Barcelona (UAB), Barcelona, Spain; ^3^Department of Electronic Engineering and Institute for Research and Innovation in Health (IRIS), Universitat Politècnica de Catalunya, Barcelona, Spain; ^4^Department of Internal Medicine, Hospital Universitari Germans Trias i Pujol, Barcelona, Spain; ^5^Department of Infectious Diseases, Hospital Universitari Germans Trias i Pujol, Barcelona, Spain; ^6^Department of Nephrology, Hospital Universitari Germans Trias i Pujol, Barcelona, Spain

**Keywords:** COVID-19, Hospital-at-Home program, biomarkers, Hs-TnT, machine learning

## Abstract

**Objectives:**

During the coronavirus disease 2019 (COVID-19) pandemic, the Hospital-at-Home (HaH) program played a key role in expanding healthcare capacity and managing COVID-19 pneumonia. This study aims to evaluate the factors contributing to readmission from HaH to conventional hospitalization and to apply classification algorithms that support discharge decisions from conventional hospitalization to HaH.

**Methods:**

Blood biomarkers (IL-6, Hs-TnT, CRP, ferritin, and D-dimer) were collected from 871 patients transferred to HaH after conventional hospitalization for COVID-19 at the *Hospital Universitari Germans Trias i Pujol*. Of these, 840 patients completed their recovery without any complications, while 31 of them required readmission. Statistical tests were conducted to assess differences in blood biomarkers between the first day of conventional hospitalization and the first day of HaH, as well as between patients who successfully completed HaH and those who were readmitted. Various classification algorithms (bagged trees, KNN, LDA, logistic regression, Naïve Bayes, and the support vector machine [SVM]) were implemented to predict readmission, with performance evaluated using accuracy, sensitivity, specificity, F1 score, and the Matthews Correlation Coefficient (MCC).

**Results:**

Significant differences were observed in IL-6, Hs-TnT, CRP (*p* < 0.001), and ferritin (*p* < 0.01) between the first day of conventional hospitalization and the first day of HaH for patients who were not readmitted. However, no significant differences were found in patients who were readmitted. At HaH, readmitted patients exhibited higher CRP and Hs-TnT values. Among the classification algorithms, the SVM showed the best performance, achieving 85% sensitivity, 87% specificity, 86% accuracy, 84% F1 score, and 71% MCC.

**Conclusion:**

Hs-TnT was a key predictor of readmission for COVID-19 patients discharged to HaH. Classification algorithms can aid clinicians in making informed decisions regarding patient transfers from conventional hospitalization to HaH.

## 1 Introduction

At the end of 2019, the COVID-19 pandemic placed immense strain on hospitals worldwide, as a surge of affected people required admission to conventional hospital settings. This unprecedented situation pushed healthcare systems to their capacity limits, requiring rapid, temporary expansions to accommodate the increasing demand for hospital care (1). Discharging patients based on clinical criteria from conventional hospitalization to Hospital-at-Home (HaH) emerged as a crucial strategy to free up hospital beds for more critically ill patients while ensuring that discharged patients continued receiving hospital-level care in their homes (2, 3). HaH is an acute hospital substitution service that allows patients to receive treatment and hospital-level care in the comfort of their own homes, with conditions comparable to those of conventional hospital stays.^1^

Multiple studies evaluating the HaH unit conclude that it is both safe and effective in optimizing hospital resources and capacity, especially during the pandemic (1–4). Various studies have shown that HaH is a safe and effective alternative for managing COVID-19 patients with varying levels of severity, allowing for better allocation of hospital resources. In all studies, the inclusion criteria for HaH candidates were based on clinical, basic analytical, or respiratory function parameters. Additionally, clinical protocols and treatments were adjusted throughout the different waves of the pandemic to align with changing therapeutic guidelines (1).

During the first four waves and amid various COVID-19 variants, the “*Hospital Universitari Germans Trias i Pujol*” treated 3,038 patients, of whom 871 patients (28.6%) were discharged through HaH to reduce their hospital stay. Additionally, 3.6% of patients were readmitted due to poor clinical progress. Although the results were very good based on the selection criteria used, we believe that developing clinical-analytical algorithms for patient selection can enhance the quality of care provided in the HaH and improve clinical safety.

The various studies published show little variation among them regarding the clinical criteria for discharging COVID-19 patients. Among the majority of frequently mentioned characteristics are suitable home conditions, the absence of fever, a good respiratory rate, and adequate oxygen saturation (1). In cases of COVID-19 pneumonia, in addition to the clinical and analytical criteria described above, different biomarkers are associated with disease severity or progression (including CRP, IL-6, D-DIMER, and Hs-TnT) or mortality (D-DIMER and Hs-TnT) as well (5–10). Although these biomarkers are not systematically used in clinical practice for decision-making, it is reasonable to consider that they can help identify patients who require admission, those at risk of progression to invasive ventilation, and high-risk patients who require special treatments. In our case, these can assist in making the decision to transfer the patient to HaH with maximum safety, thereby avoiding readmissions to conventional hospitalization.

Generally, large datasets lead to better classification performance, while smaller datasets may cause overfitting (when the algorithm fits the training data well but fails to generalize). However, in practice, collecting medical data presents challenges due to patient privacy concerns, the rarity of certain conditions, and various organizational and legal obstacles (11). To address these difficulties, data augmentation algorithms provide a way to create additional data samples for effective model training. This method is valuable for extracting richer insights from limited data. Several researchers have implemented data augmentation techniques to increase the diversity of their datasets (12).

There are various algorithms for data augmentation, with their application depending on the data type. For quantitative data, one prominent algorithm is the Synthetic Minority Over-sampling Technique (SMOTE). SMOTE is designed to enhance the performance of machine learning algorithms by generating new data through oversampling the minority class, which aims to improve classification accuracy. This method identifies samples that are close to each other (k-nearest neighbors) in the feature space, draws a line between these samples, and adds a new synthetic sample at a point along this line (13, 14).

Regarding the application of machine learning (ML), it classifies new data points (or patients) into previously defined groups based on a trained model. Thus, ML presents an opportunity to help clinicians in decision-making. There are multiple ML algorithms, including bagged trees, K-nearest neighbors (KNN), linear discriminant analysis (LDA), logistic regression, Naïve Bayes, and support vector machines (SVMs) (15). A decision tree algorithm involves building a hierarchy of if/else questions that lead to a decision. In bagging, multiple decision trees are constructed before a decision is made through majority voting. Bagging enhances the accuracy and robustness of the decision tree. KNN predicts the class of an unseen sample using the information from the K-nearest neighbors based on feature similarity. LDA finds a linear combination of features that best separates different classes of data. Logistic regression predicts the likelihood of a binary output using one or more input features by applying a logistic (or sigmoid) function to convert predictions into probabilities, which are then utilized for classification. Naïve Bayes models learn by considering each feature independently and collecting simple statistics for each class. For our application, Gaussian Naïve Bayes is used as it handles continuous data effectively, unlike binary or count data. Finally, the SVM algorithm aims to identify the best hyperplane that divides the data points of different classes. This hyperplane is chosen to maximize the margin, which is the distance between the hyperplane and the closest data points from each class (16, 17).

The evaluation of the algorithms’ classification tasks is conducted using performance metrics such as sensitivity, specificity, F1 score, and accuracy. In addition, for unbalanced datasets, the Matthews Correlation Coefficient (MCC) serves as the evaluation metric (18).

Considering all the points mentioned above, this study aims to evaluate the factors that lead to the readmission of COVID-19 patients who develop pneumonia from HaH to conventional hospitalization. In addition, it seeks to validate, using machine learning techniques, the usefulness of a set of biomarkers (IL-6, Hs-TnT, CRP, ferritin, and D-dimer) registered on the first day of HaH admission and their role in the readmission of patients admitted to HaH for COVID-19.

## 2 Materials and methods

### 2.1 Participants

Blood biomarkers (IL-6, Hs-TnT, CRP, ferritin, and D-dimer) were collected from a total of 871 patients (age: 59 ± 15 years; weight: 81.5 ± 18.5 kg; BMI: 29.3 ± 9.2 kg/m^−2^) who were transferred to HaH after being conventionally hospitalized at the “*Hospital Universitari Germans Trias i Pujol*” (HUGTiP) for COVID-19 between March 2020 and July 2022.

### 2.2 Study protocol

This is an observational retrospective study in which blood biomarkers (IL-6, Hs-TnT, CRP, ferritin, and D-dimer) were collected immediately before referral to HaH. The patients referred to HaH after conventional hospitalization included those with COVID-19 who required hospitalization but could be treated at home due to their good clinical and analytical progress, thereby reducing the duration of conventional hospitalization. Of the 2,839 patients with COVID-19 pneumonia admitted to the hospital (conventional hospitalization, semi-critical, and ICU), 871 patients (30.68%) were transferred to the HaH Unit to decrease the average stay, enable home admission, and free up hospital beds during a time of great need. The criteria for transfer to HaH include fulfilling the following conditions: a heart rate of <100 bpm, a respiratory rate of <24 rpm, an axillary temperature of <37.2°C, a systolic blood pressure of >90 mmHg, a basal oxygen saturation of >90% (if there was no previous respiratory failure), and an adequate level of consciousness.

These criteria had to be accompanied by an analytical improvement (a decrease in CRP, LDH, and transaminases and resolution of leukopenia), which was evaluated based on medical judgment. In addition, patients were transferred to HaH only if their socio-familial and home conditions were deemed suitable. Each case was individually evaluated and managed in accordance with public health guidelines.

The protocol for home monitoring of patients was the same for all patients and included at least one daily medical and/or nursing visit, telemedicine monitoring, and complementary examinations according to healthcare requirements. Patients receiving care at home could access oxygen therapy, nebulizations, and intravenous antibiotic or antiviral treatment as needed.

Patients exhibiting poor progress, defined by persistent fever, worsening respiratory symptoms, and oxygen saturation levels below 90–92% based on individual cases, were readmitted to the conventional hospital.

### 2.3 Data analysis

#### 2.3.1 Statistical analysis

The chi-square test was used to evaluate differences in previous pathologies between patients who successfully performed HaH and those readmitted to conventional hospitalization after being home hospitalized. The Shapiro–Wilk test was used to assess the normality of variable distribution (IL-6, troponin, CRP, ferritin, and D-dimer) for patients readmitted to conventional hospitalization, while the Kolmogorov–Smirnov test was used to evaluate normal distribution for the group of patients succeeding in HaH. Variables that were not normally distributed are presented as median (interquartile range, IQR) and minimum–maximum. Normally distributed variables are represented as mean ± SD with a 95% confidence interval (CI) (lower bound–upper bound). The Wilcoxon test was applied to determine differences in blood biomarkers between values on the first day of conventional hospitalization and the first day of HaH. The Mann–Whitney U test was used to assess the significance of blood biomarkers between patients succeeding in HaH and those readmitted to conventional hospitalization after being home hospitalized.

The statistical software IBM®SPSS®version 24.0 (IBM Corp, Armonk, NY, United States) was used for data analysis. The level of statistical significance was set at *p* < 0.05.

#### 2.3.2 Data augmentation

From the 871 patients initially gathered, only those with complete information about all the biomarkers collected (IL-6, Hs-TnT, CRP, ferritin, and D-dimer) were included in the study. The final number of patients was 779, consisting of 755 patients who successfully ended recovery at HaH and 24 patients who needed to be readmitted to conventional hospitalization from HaH. The SMOTE technique for data augmentation was applied to the minority group (patients requiring readmission to conventional hospitalization). An oversampling of 1,000% was conducted, increasing the number of samples from 24 original data points to 240 synthetic values, resulting in a final sample size of 264 patients for the minority group. A maximum of 10 nearest neighbors was selected to minimize bias. Finally, of the 755 patients who completed recovery at HaH successfully, 264 were randomly selected for the study to balance the groups. The dataset used for analysis consisted of 528 patients (rows) and five input variables (columns) representing biomarkers: IL-6, Hs-TnT, CRP, ferritin, and D-dimer.

#### 2.3.3 Machine learning algorithms

A model-blind test set was created to apply classification algorithms, which is separate from the training and cross-validation sets. To minimize bias in the test set selection, the data was randomly shuffled. A total of 20% of the data were set aside as a blind test set, while 80% of the data were used to perform K-Fold Cross-validation (KCV) with five folds. Cross-validation was used as a strategy to reduce data overfitting.

Hyperparameter optimization during the KCV process was performed to select the parameters to achieve the highest possible accuracy. As a binary classification problem, typical algorithms used for this purpose included logistic regression and the SVM. Additionally, LDA, bagged trees, KNN, and Naïve Bayes were implemented, although these also accommodate multiclass classification problems. To evaluate the performance of each algorithm, the accuracy ± standard deviation of accuracy across the five folds was calculated. The majority of suitable algorithm for the data was then used to classify the blind test set. The following metrics were calculated to evaluate the performance of the best algorithm: accuracy, sensitivity, specificity, F1 score, and MCC.

The software MATLAB version 23.2.0.2485118 (R2023b), Natick, Massachusetts: The MathWorks Inc.; 2023, has been used to implement the algorithms with the Classification Learner App.

## 3 Results

**Figure 1:**
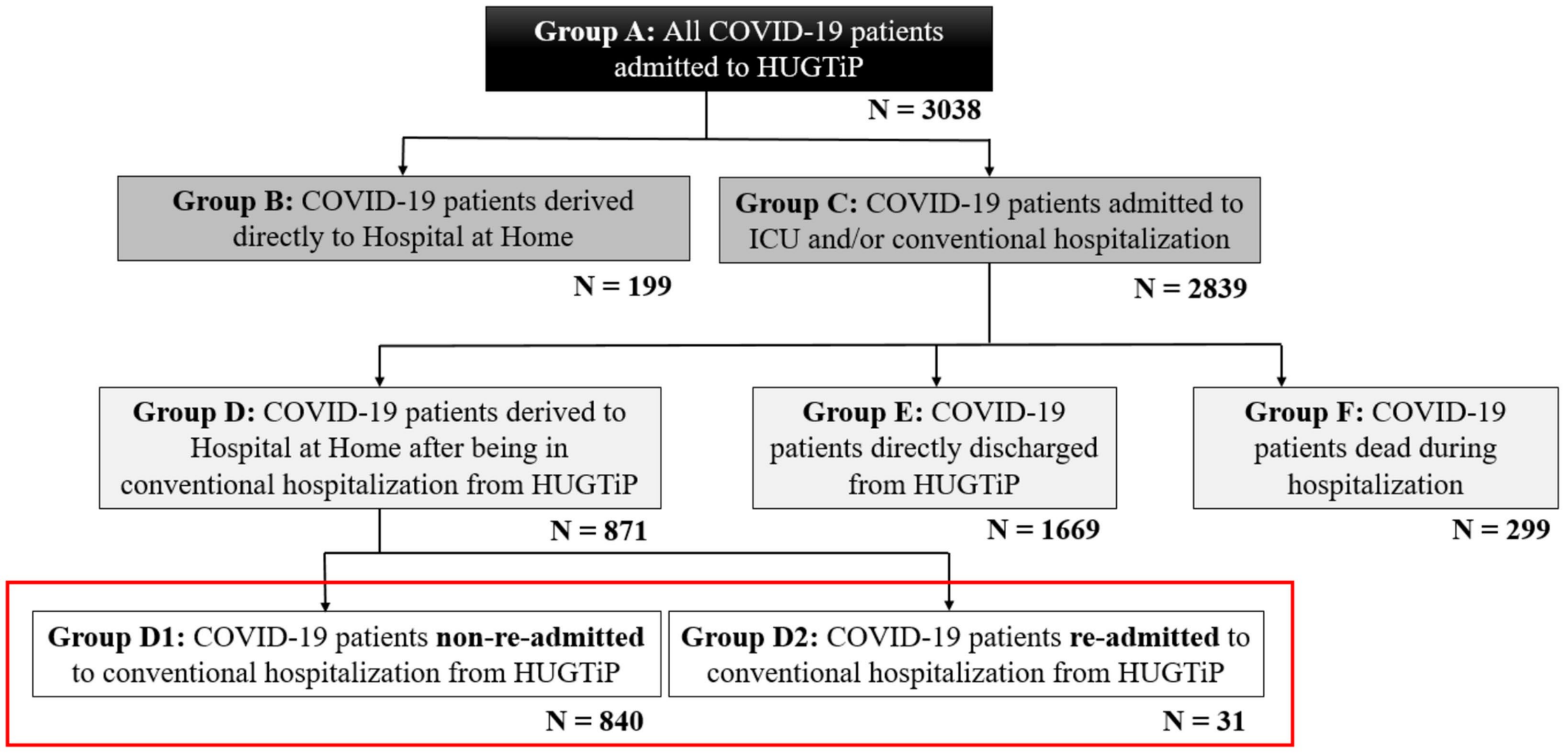
Distribution of COVID-19 patients admitted to the *Hospital Universitari Germans Trias i Pujol.*

Figure 1 shows the distribution of patients who went to “*Hospital Universitari Germans Trias i Pujol*.” A total of 3,038 patients (Group A) visited “*Hospital Universitari Germans Trias i Pujol*” for COVID-19 (age: 61 ± 17 years; weight: 79.9 ± 18.5 kg; BMI: 29.0 ± 10.2 kg/m^−2^). Of the 3,038 patients, 199 of them (Group B) did not require hospitalization and were directed straight to HaH (age: 56 ± 17 years; weight: 69.5 ± 14.8 kg; BMI: 25.2 ± 4.9 kg/m^−2^), whereas 2,839 (Group C) required conventional hospitalization. Among the patients needing conventional hospitalization, 871 (Group D) were referred to HaH to complete their recovery (age: 59 ± 15 years; weight: 81.5 ± 18.5 kg; BMI: 29.3 ± 9.2 kg/m^−2^), while 1,669 of them (Group E) were discharged directly from conventional hospitalization (age: 61 ± 17 years; Weight: 80.2 ± 18.3 kg; BMI: 29.1 ± 10.3 kg/m^−2^). Unfortunately, 299 patients (Group F) died during conventional hospitalization (age: 75 ± 12 years; weight: 76.3 ± 19.1 kg; BMI: 28.8 ± 12.6 kg/m^−2^). Finally, among the 871 patients transferred to HaH to complete their recovery, 840 of them (Group D1) reported no complications during recovery (age: 60 ± 15 years; weight: 82.0 ± 18.7 kg; BMI: 29.4 ± 9.3 kg/m^−2^), while 31 of them (Group D2) required readmission to HaH (age: 71 ± 15 years; weight: 72.2 ± 12.5 kg; BMI: 27.2 ± 4.6 kg/m^−2^). A red square is used to visualize the groups analyzed in this study.

Blood biomarkers (IL-6, Hs-TnT, CRP, ferritin, and D-dimer) were collected on the first day of conventional hospitalization and immediately before referral to HaH, involving a total of 871 patients. Among these, 31 patients from HaH were readmitted to conventional hospitalization, with a readmission duration of 7 ± 6 days, while the total number of patients discharged from HaH was 840.

### 3.1 Descriptive analysis of previous patients’ conditions

Table 1 shows the descriptive analysis of prior pathologies experienced by all COVID-19 patients (Group A), as well as individual analyses for Groups D and E. It also includes a descriptive analysis of subgroup D regarding prior pathologies in Groups D1 and D2. Furthermore, the Chi-Square test results, including the statistic *χ*^2^ and the significance *p*-value, are displayed for the comparison of prior pathologies between Groups D and E and between Groups D1 and D2. Group A (all COVID-19) consists of patients who visited HUGTiP for COVID-19; Group D includes patients referred to HaH after conventional hospitalization for COVID-19; Group E comprises patients directly discharged from conventional hospitalization following treatment for COVID-19; Group D1 (non-readmitted) refers to patients from HUGTiP who were sent to HaH after conventional hospitalization and successfully completed their recovery; Group D2 (readmitted) refers to patients from HUGTiP who were referred to HaH after conventional hospitalization and required readmission to conventional care.

**Table 1:** Percentage of patients with previous pathologies who visited “*Hospital Universitari Germans Trias i Pujol*” due to COVID-19.

	Group A *N* = 3,038 (%)	D *N* = 871 (%)	E *N* = 1,669 (%)	*χ* ^2^	*p*
Asthma	7.4	7.1	7.5	0.155	0.694
Diabetes I	1.1	1	1.1	0.058	0.810
Diabetes II	30.7	29.3	30.1	0.176	0.675
Arterial Hypertension	43.5	42.9	41.9	0.262	0.608
COPD	10.7	10.2	9.8	0.098	0.754
Obesity	43.2	45.4	44.2	0.329	0.566
Ischemic cardiopathy	18.9	17.3	17.9	0.131	0.717
Heart failure	9.4	5.5	10.0	14.925	<0.001
Dyslipidemia	51.5	51.9	49.4	1.458	0.227
Renal insufficiency	16.1	11.7	16.3	9.588	0.002

	Group A *N* = 3,038 (%)	D1 *N* = 840 (%)	D2 *N* = 31 (%)	*χ* ^2^	*p*
Asthma	7.4	7.14	6.45	0.022	0.883
Diabetes I	1.1	0.95	3.23	1.511	0.219
Diabetes II	30.7	28.33	54.84	10.144	0.001
Arterial hypertension	43.5	42.14	64.52	6.108	0.013
COPD	10.7	9.64	25.81	8.514	0.004
Obesity	43.2	45.48	41.94	0.151	0.697
Ischemic cardiopathy	18.9	16.79	32.26	4.994	0.025
Heart failure	9.4	4.76	25.81	25.427	<0.001
Dyslipidemia	51.5	50.95	77.42	8.389	0.004
Renal insufficiency	16.1	10.71	38.71	22.662	<0.001

In addition, the results of the Chi-Square statistical test and the statistic *χ*^2^ compare the pathologies of patients between groups D and E, as well as between groups D1 and D2.

The majority of common causes of readmission to conventional hospitalization in D2 groups included fever (*N* = 5, 16.13%), dyspnea (*N* = 13, 41.94%), worsening respiratory conditions (*N* = 4, 12.90%), COVID-19 pneumonia (*N* = 5, 16.13%), and worsening of general conditions (*N* = 8, 25.81%).

### 3.2 Differences in blood biomarkers between the first day of conventional hospitalization and the first day of HaH

**Figure 2:**
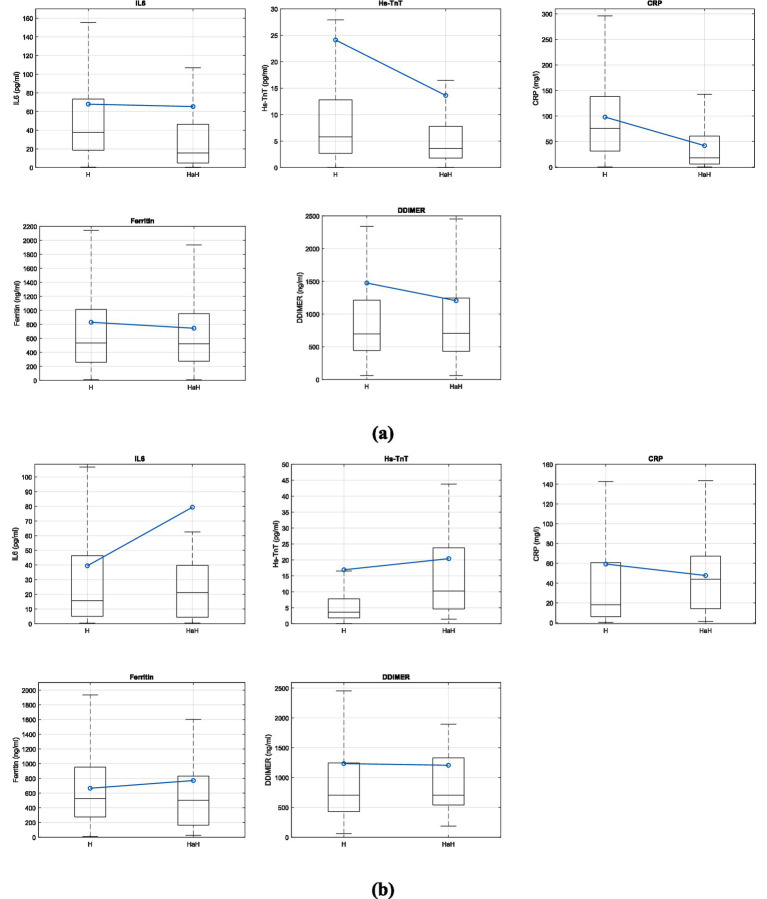
Boxplot representation of the variables IL6, Hs-TnT, CRP, Ferritin and DDIMER the first day of conventional hospitalization and the first day of hospital at home for **(a)** D1 group and **(b)** D2 group.

Figure 2 shows the boxplot representation of blood biomarker values on the first day of conventional hospitalization and the first day of HaH for (a) D1 and (b) D2 groups. In each box, the center mark indicates the median, while the bottom and top ends represent the 25th and 75th percentiles, respectively. Whiskers extend to the most extreme data points that are not considered outliers. The blue line represents the mean values at each moment. Outliers are not shown for visualization purposes.

Table 2 shows the description of the IL-6, Hs-TnT, CRP, ferritin, and D-dimer variables on the first day of conventional hospitalization (H) and the first day of HaH, divided according to non-readmitted (D1) and readmitted (D2) patients. In addition, the statistic of W of the Wilcoxon test for non-parametric related samples and the statistical significance of the test are also represented.

**Table 2:** Description of the IL-6, Hs-TnT, CRP, ferritin, and D-dimer variables.

	Descriptive		*W*	*p*
	D1			
	H	HaH		
IL-6	39.17 (55.62) (0.42–1,632)	16.04 (43.21) (0.37–1,632)	−10.360	<0.001
Hs-TnT	6 (10.8) (0–3342.7)	3.6 (6) (0–917.7)	−14.583	<0.001
CRP	78.8 (107.7) (0.3–666.4)	17.4 (51.1) (0.3–466.2)	−17.538	<0.001
Ferritin	548.1 (792) (12–12,148)	530 (673) (16–12,148)	−2.963	0.003
D-DIMER	712 (777) (59–131,129)	705 (826) (59–16,397)	−1.588	0.112
	D2			
	H	HaH		
IL-6	24.8 (26.68) (1.51–139.43)	18.01 (42.07) (0.4–1425.79)	−1.013	0.311
Hs-TnT	10.3 (15.23) (1.4–60)	10.25 (24.28) (1.4–86.8)	0.000	1.000
CRP	44.6 (58.25) (7.5–281.1)	43.9 (50.6) (1.2–131.1)	−1.232	0.218
Ferritin	405.5 (619.85) (26–4,470)	439.5 (713.48) (26–4,470)	2.045	0.041
D-dimer	690 (946) (249–4,645)	695.5 (792) (186–4,645)	−0.622	0.534

In addition, the Wilcoxon statistic (W) and the statistical significance are also shown. The variables that are not normally distributed are shown as the median (interquartile range, IQR) and minimum-maximum values. Normally distributed variables are represented as the mean ± SD with a 95% CI (lower bound–upper bound).

### 3.3 Inter-subject differentiation in blood biomarkers

**Figure 3:**
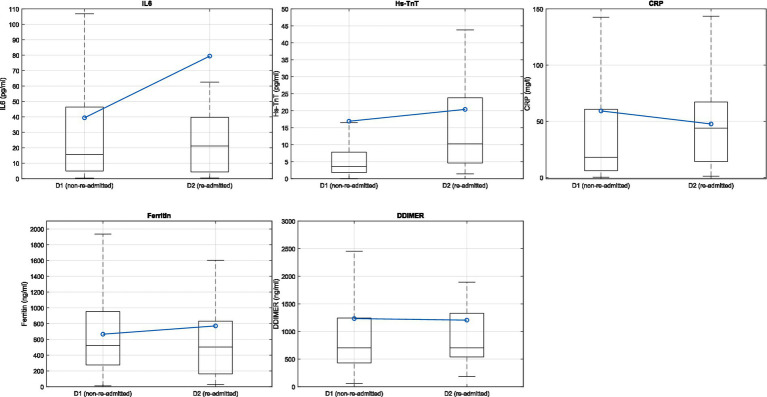
Boxplot representation of the variables IL-6, Hs-TnT, CRP, ferritin, and D-dimer at HaH for non-readmitted (D1) and readmitted (D2) patients.

Figure 3 shows a boxplot comparing the IL-6, Hs-TnT, CRP, ferritin, and D-dimer variables between non-readmitted (D1) and readmitted (D2) patients prior to their HaH admission. The central line of the box represents the median of the variables, while the lower and upper lines indicate the lower and upper quartiles, respectively. The extreme lines denote the minimum and maximum values that are not considered outliers. Outliers are excluded for clarity. The blue line illustrates the mean values for each group.

Table 3 provides descriptions of the IL-6, Hs-TnT, CRP, ferritin, and D-dimer variables, categorized by non-readmitted (D1) and readmitted (D2) patients. Additionally, the results of the Mann–Whitney U test and the statistical significance of the analyses are also included.

**Table 3:** Description of IL-6, Hs-TnT, CRP, ferritin, and D-dimer.

	Descriptive			
	D1 (Non-readmitted)	D2 (Readmitted)	*U*	*p*
IL-6	16.04 (43.21) (0.37–1,632)	18.01 (42.07) (0.4–1425.79)	−0.182	0.856
Hs-TnT	3.6 (6) (0–917.7)	10.25 (24.28) (1.4–86.8)	4.035	<0.001
CRP	17.4 (51.1) (0.3–466.2)	43.9 (50.6) (1.2–131.1)	1.965	0.049
Ferritin	530 (673) (16–12,148)	439.5 (713.48) (26–4,470)	−0.801	0.423
D-dimer	705 (826) (59–16,397)	695.5 (792) (186–4,645)	0.707	0.479

In addition, the Mann–Whitney U statistic and its statistical significance are also included. Non-normally distributed variables are indicated as median statistics (interquartile range, IQR), along with minimum and maximum values. Normally distributed variables are expressed as mean ± SD with a 95% CI (lower bound–upper bound).

### 3.4 Data augmentation

**Figure 4:**
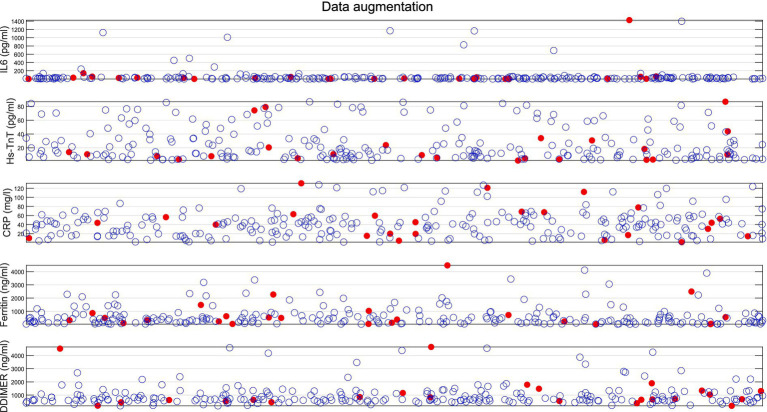
Results of data augmentation for patients returning to conventional hospitalization from HaH. Red dots represent original data values, while blue circles indicate the synthetic data created.

Figure 4 shows the results of data augmentation using the SMOTE process for all the biomarkers analyzed (IL-6, Hs-TnT, CRP, ferritin, and D-dimer) in the implementation of the machine learning classification algorithms. The red dots indicate the original values from patients who returned to conventional hospitalization after being discharged to HaH, while the blue circles represent the synthetic data generated.

Table 4 shows the descriptive and statistical analysis of the original data compared to SMOTE + the original data for all the biomarkers used (IL-6, Hs-TnT, CRP, ferritin, and D-dimer), showing no significant differences (*p* > 0.05) between the two groups.

**Table 4:** Description of IL-6, Hs-TnT, CRP, ferritin, and D-dimer for both the original data and the SMOTE + Original data.

	Descriptive		*U*	*p*
	D2			
	Original data	SMOTE + Original data		
IL-6	18.01 (42.07) (0.4–1425.79)	20.50 (32.64) (0.4–1425.79)	0.409	0.682
Hs-TnT	10.25 (24.28) (1.4–86.8)	17.54 (25.93) (1.4–86.8)	1.677	0.094
CRP	43.9 (50.6) (1.2–131.1)	41.58 (37.29) (1.2–131.1)	−0.036	0.971
Ferritin	439.5 (713.48) (26–4,470)	321.25 (496.95) (26–4,470)	−0.864	0.387
D-dimer	695.5 (792) (186–4,645)	690.13 (494.56) (186–4,645)	−0.514	0.607

In addition, the U of Mann–Whitney statistic and its statistical significance are included. The variables that are non-normally distributed are represented as the median statistic (interquartile range, IQR), along with minimum and maximum values.

### 3.5 Machine learning algorithms classification results

Table 5 shows the accuracy, misclassification cost, and the AUC obtained during the 5-fold cross-validation process for the multiple algorithms tested (bagged trees, KNN, LDA, logistic regression, Naïve Bayes, and the SVM).

**Table 5:** Accuracy, misclassification cost, and the AUC obtained during the 5-fold cross-validation process for the tested models (bagged trees, KNN, LDA, logistic regression, Naïve Bayes, and the SVM).

Model	Accuracy	Misclassification cost	AUC
Bagged trees	85.3	62	0.93
KNN	84.4	66	0.84
LDA	71.1	122	0.73
Logistic regression	73.7	111	0.75
Naïve Bayes	78.2	92	0.82
SVM	86.7	56	0.91

**Figure 5:**
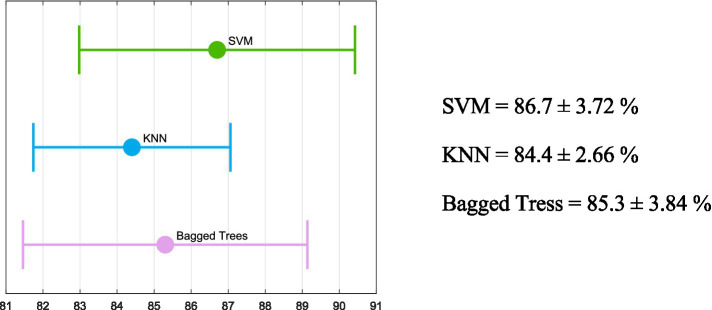
Mean and standard deviation obtained from the 5-fold cross-validation process for the algorithms with an accuracy above 80% (bagged trees, KNN, and the SVM).

Figure 5 shows the mean accuracy values obtained through the 5-fold cross-validation process, along with the standard deviation for the algorithms that achieved a mean accuracy above 80%. The SVM produced the best results; therefore, it is used to classify new blind-test data.

**Figure 6:**
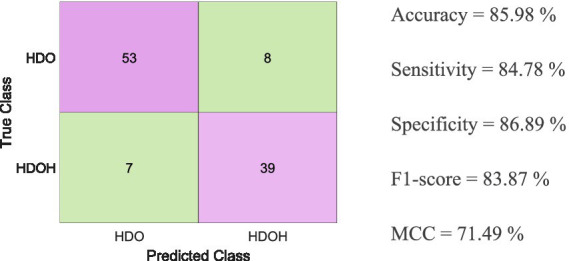
Classification metrics for the blind test data using the SVM-trained model, along with the confusion matrix showing accuracy, sensitivity, specificity, F1 score, and MCC obtained.

Figure 6 shows the classification metrics for the blind test data using the previously trained SVM model. It shows the confusion matrix along with the accuracy, sensitivity, specificity, F1 score, and MCC achieved.

## 4 Discussion

During the COVID-19 pandemic, some patients with COVID-19 pneumonia who were in conventional hospitalization were transferred to HaH to free up beds for more critically ill patients. Although the majority of patients (840) successfully completed their treatment in HaH, 31 of them required readmission to conventional hospitalization after failing their treatment (Figure 1). According to published literature on COVID-19, patients readmitted to conventional hospitalization had a higher average age than compared to those who successfully completed HaH without readmission.

This study evaluated the differences in blood biomarkers between patients who were readmitted to conventional hospitalization (D2) and those who recovered successfully in HaH (D1).

The patients transferred to HaH had significantly more comorbidities, as reflected in Table 1, including a higher prevalence of underlying cardiorespiratory pathology (*p* < 0.001) and renal failure (*p* < 0.01). Consequently, they required medical management for all their pathologies in addition to hospitalization for COVID-19 pneumonia. Consequently, we can state that the patients directed to HaH represented a high-complexity subgroup with an extended average length of stay forecast. Many of the pathologies affecting our patients have been correlated with high mortality rates in extensive studies on COVID-19. Generally, patients with pre-existing conditions are more likely to fail the home hospitalization process (Table 1). Conditions such as Diabetes II, COPD, and dyslipidemia (*p* < 0.01), as well as arterial hypertension, ischemic cardiopathy (*p* < 0.05), and heart failure and renal insufficiency (*p* < 0.000), were more prevalent among patients who failed HaH compared to those who successfully completed the process. Given that a confounding variable is a factor that interferes with the relationship between the independent and dependent variables in a study, these comorbidities may act as potential confounders. After this analysis, we believe that part of the clinical criteria mentioned in section 2.2 and the different comorbidities that were statistically significant should be considered in future admissions of patients to HaH due to COVID-19. Furthermore, if comorbidities are integrated into the algorithms, the validation of the selected biomarker package would not be possible since the algorithms would not classify solely based on these variables but by incorporating additional factors. Asthma, obesity, and diabetes did not significantly influence the recovery process (*p* > 0.05). Overall, the results show that people readmitted to conventional hospitalization exhibited a more complex clinical history.

To ensure patient safety during admission to the HaH for the majority of critical cases (such as respiratory failure requiring oxygen at home), the use of telemedicine was proposed through a telemedicine platform and an app for two-way interaction between the healthcare team and the patient-caregiver. In addition to medical and nursing visits, telephone monitoring, and complementary tests, all COVID-19 patients were monitored for temperature and provided with a pulse oximeter to monitor oxygen saturation.

Regarding other possible confounding variables, the authors found no statistically significant differences in sex. In the D1 group, there were 326 (38.81%) women and 514 (61.19%) men; in the D2 group, there were 10 (32.26%) women and 21 (67.74%) men, with a *χ* = 0.542 and *p* = 0.462. Length of hospital stay, which had a non-normal distribution, showed statistical significance (*U* = −3.231 and *p* = 0.001), with a value of 10 (5) (5–21) for the D1 group and 7 (6) (1–18) for the D2 group. Biomarker values were collected just before admission to HaH, and the values of the variables on the first day of conventional hospitalization were not included to minimize the impact of hospitalization on these values. Regarding age, statistical significance was obtained (*F* = 18.92 and *p* < 0.001), with a normally distributed value of 64 ± 11 (60–68) for the D1 group and 71 ± 14 (66–76) for the D2 group. Studies on COVID-19 patients from various countries highlight the significant impact of age on mortality, identifying critical thresholds for ages over 50 years and especially over 60 years (19). Accordingly, when separating by age groups, we found the following: (1) patients under 50 years: 227 (27.02%) patients in D1 and 2 (6.45%) patients in D2 with a *p* = 0.011; (2) patients between 50 and 60 years: 201 (23.93%) patients in D1 and 3 (9.68%) patients in D2 with a *p* = 0.066; (3) patients between 61 and 70 years: 196 patients (23.33%) in D1 and six patients (19.35%) in D2 with a *p* = 0.606; (4) patients between 71 and 80 years: 155 patients (18.45%) in D1 and 14 patients (45.16%) in D2 with a *p* < 0.001; and (5) patients >80 years: 61 patients (7.26%) in D1 and six patients (19.35%) in D2 with a *p* = 0.013. Therefore, we found that patients returning to conventional hospitalization after HaH were statistically older than those who completed their treatment in HaH.

Biomarker values vary with age (20–23). Particularly, we found statistically significant correlations between age and IL-6 (ρ = 0.097), Hs-TnT (ρ = 0.374), and D-dimer (ρ = 0.164), with a *p*-value of <0.001. However, the correlation coefficient is less than 0.5 for all the biomarkers, meaning that age could not be considered as a confounding variable. Moreover, although not directly, age is already incorporated into the algorithm. Incorporating correlated variables in machine learning algorithms reduces classification metrics (24, 25) while introducing redundancy, which does not contribute additional knowledge to the algorithm but increases model complexity. Furthermore, removing correlated features enhances generalization capacity. For this reason, age has been excluded from the algorithm. Moreover, the study aims to validate, using machine learning techniques, the usefulness of a set of biomarkers without accounting for other variables.

For all these reasons, we thought it was convenient to look for markers that would allow us to identify patients who required hospital readmission. According to Bhattacharyya et al. (26), the selected package of biomarkers is categorized as predictive, as the goal is to determine the treatment response (transitioning from conventional hospitalization to HaH) while the disease (COVID-19) remains present. Surprisingly, given the patient’s profile and using only the standard selection criteria, the results were very good. However, despite this, a percentage of patients were readmitted to conventional hospitalization.

We selected five markers clearly related to the mortality and severity of COVID-19 that were not included in the initial discharge protocols to determine whether we could successfully identify patients who were candidates for transfer to the HaH.

Regarding the results shown in Figure 2 and Table 2, it is important to note that patients who did not require readmission to conventional hospitalization (D1) exhibited greater changes in the analyzed blood biomarkers, with significant differences observed in IL-6, Hs-TnT, CRP (*p* < 0.000), and ferritin (*p* < 0.01). However, no significant differences are found in blood biomarkers between the first day of conventional hospitalization and the first day of HaH for patients who needed readmission to conventional hospitalization (D2), particularly with respect to Hs-TnT, where the significance (P) is 1. Therefore, it is crucial to evaluate the patient’s evolution rather than focus solely on the blood biomarker values at the end of conventional hospitalization. Moreover, although protocols were accurate for the majority of patients, complex cases (such as those referred to HaH) require thorough evaluation, and protocols must be tailored to each individual.

With respect to the results obtained from differentiating between groups D1 and D2 in blood biomarkers (Figure 3; Table 3), the Mann–Whitney U statistical test was conducted to evaluate differences in blood biomarkers (IL-6, Hs-TnT, CRP, ferritin, and D-dimer) between D1 (non-readmitted) and D2 (readmitted) patients. The results indicated statistical differences in Hs-TnT and CRP (*p* < 0.05). This finding reveals that patients who were readmitted to conventional hospitalization had higher levels of Hs-TnT compared to those who did not require readmission, suggesting that the cardiac muscle of readmitted patients was more damaged than that of non-readmitted patients.

The HaH unit gained importance during the COVID-19 pandemic, particularly at a time when there were not enough beds available to accommodate patients. This situation marked an inflection point for the HaH unit, which has continued to grow in significance since then. Although the vast majority of patients (96.44%) recover successfully in the HaH unit, 3.56% of patients needed to be readmitted to conventional hospitalization. This indicates that while clinical protocols were effective, a small percentage still did not succeed. For this reason, it is important to develop tools to assist clinicians in their decision-making, such as determining whether it is appropriate to discharge a patient from conventional hospitalization to the HaH unit.

One limitation of clinical studies is the low availability of data, as seen in this study, which includes only 24 cases from the minority group for analysis. On the one hand, this reflects the accuracy of clinical protocols. On the other hand, to address the issue of unbalanced and scarce data, it is necessary to apply data augmentation techniques to improve the accuracy and training of the algorithm. The SMOTE algorithm for data augmentation has been used to increase the balance between the group discharged from conventional hospitalization to HaH and successfully completed recovery, as well as the group that required readmission to conventional hospitalization. The drawback of applying these types of algorithms is that if there are biases in the data, the algorithm will also generate synthetic points near those biased values, further exacerbating the bias. To address such situations, instead of using the default five neighbors, we selected 10 nearest neighbors when generating new data points from the 24 samples in the minority group (those requiring readmission from HaH to conventional hospitalization). This adjustment aimed to minimize bias as much as possible. Data visualization helps analyze the synthetic samples to ensure their validity and that they fall within the feature range of the minority class (27). According to the results of the data augmentation algorithm shown in Figure 4, where red points represent real data and blue points denote synthetic samples, it has been proven that the data augmentation algorithm is effective, as all synthetic data created is close to the real samples. In addition, Table 4 shows no statistically significant differences between the original data and the SMOTE + Original data, proving that the generated data remains consistent with the original D2 dataset. In addition, when considering 10 neighbors, the influence of outer values is reduced.

The algorithm has successfully increased the sample size from the original 24 samples to 240 synthetic points, resulting in a final sample size of 264 for the minority group. From the 755 patients in the majority group (patients who were discharged to HaH from conventional hospitalization and completed recovery successfully), we randomly selected 264 to train the algorithm (≈ 35% of the dataset). Therefore, the final size of the dataset for the algorithm comprises 528 samples and five features or biomarkers (IL-6, Hs-TnT, CRP, ferritin, and D-dimer).

We implemented a model-blind test set strategy by partitioning the data into 80% for training and 20% for testing. For the 80% allocated for training, we utilized K-Fold cross-validation with five folds. K-Fold cross-validation ensures that the model is evaluated on data it has not encountered during training in each fold. This approach prevents the model from being evaluated solely on the training data, which could result in overly optimistic performance estimates. Furthermore, cross-validation is commonly used for hyperparameter tuning. Evaluating the model across multiple splits helps establish parameters that generalize well, thus minimizing the risk of overfitting to specific datasets or configurations (27). Various algorithms (bagged trees, KNN, LDA, logistic regression, Naïve Bayes, and the SVM) have been optimized, trained, and tested to determine which algorithm is best suited for our application.

Table 5 shows the accuracy, misclassification cost, and the AUC obtained during the 5-fold cross-validation process for the multiple methods tested. As shown in Table 5 and Figure 5, bagged trees, KNN, and the SVM are the only algorithms that achieve an accuracy exceeding 80%. Furthermore, the misclassification costs—interpreted as penalties for classification errors—are 62, 66, and 56, respectively (28). The two algorithms with the area under the curve (AUC) greater than 90% are bagged trees and the SVM (92.3 and 91.5, respectively). In addition, the literature suggests that the SVM is among the best-performing algorithms in the medical field (29–31). As illustrated in Figure 5, bagged trees, KNN, and the SVM achieved a mean accuracy above 80% across the five folds. The standard deviation is also shown to ensure consistent training. In all three algorithms, the standard deviation of accuracy is low, indicating consistency. However, the SVM shows the best metrics, with the highest accuracy and the highest upper and lower bounds. For these reasons, the SVM has been chosen as the most appropriate algorithm to evaluate the blind test set data given the trained model. As depicted in Figure 6, various metrics have been obtained to assess the trained SVM model on the blind test data (accuracy, sensitivity, specificity, F1 score, and MCC). For visualization, the confusion matrix is also included. Utilizing metrics sensitive to imbalanced data, such as sensitivity and F1 score, provides a deeper understanding of model performance than accuracy alone, which can be misleading in imbalanced datasets (17).

Among the metrics used to evaluate the performance of different models, we particularly focus on sensitivity, which emphasizes the capacity to evaluate positive instances, specifically patients who will require readmission to conventional hospitalization, and scored a value of ≈85%.

The MCC, a specific case of the Pearson Correlation Coefficient (32), can be evaluated according to the criteria of the Pearson Correlation Coefficient, which states that a value of 70% or higher indicates a strong positive relationship. According to the MCC result obtained for the SVM (71.5%) shown in Figure 6, the MCC value, along with the sensitivity and accuracy values (≈ 85%), suggests that the SVM could be utilized to predict the readmission of COVID-19 patients from HaH.

This study reveals that the value of the biomarker is important not only at the time of discharge to HaH but also in the evolution of patient biomarkers from the first day of conventional hospitalization. Furthermore, among all the biomarkers, Hs-TnT appears to be the most influential factor in patient readmission.

This study also addressed the issue of unbalanced datasets by generating effective synthetic data. In addition, it predicted which COVID-19 patients transitioned from conventional hospitalization to HaH and would require readmission to conventional hospitalization, using high metrics and the SVM while considering only the blood biomarkers registered on the first day of HaH. These results open up the possibility of applying classification algorithms across various hospital services to help clinicians make decisions.

## 5 Conclusion

In conclusion, Hs-TnT emerged as the most influential biomarker associated with readmission to conventional hospitalization in COVID-19 patients discharged to HaH. In addition, ML algorithms can serve as valuable tools to help clinicians make decisions regarding patient discharge. Moreover, the challenge posed by the limited number of cases that clinicians often encounter can be effectively addressed through the application of data augmentation algorithms.

## 6 Contribution

**Figure 7:**
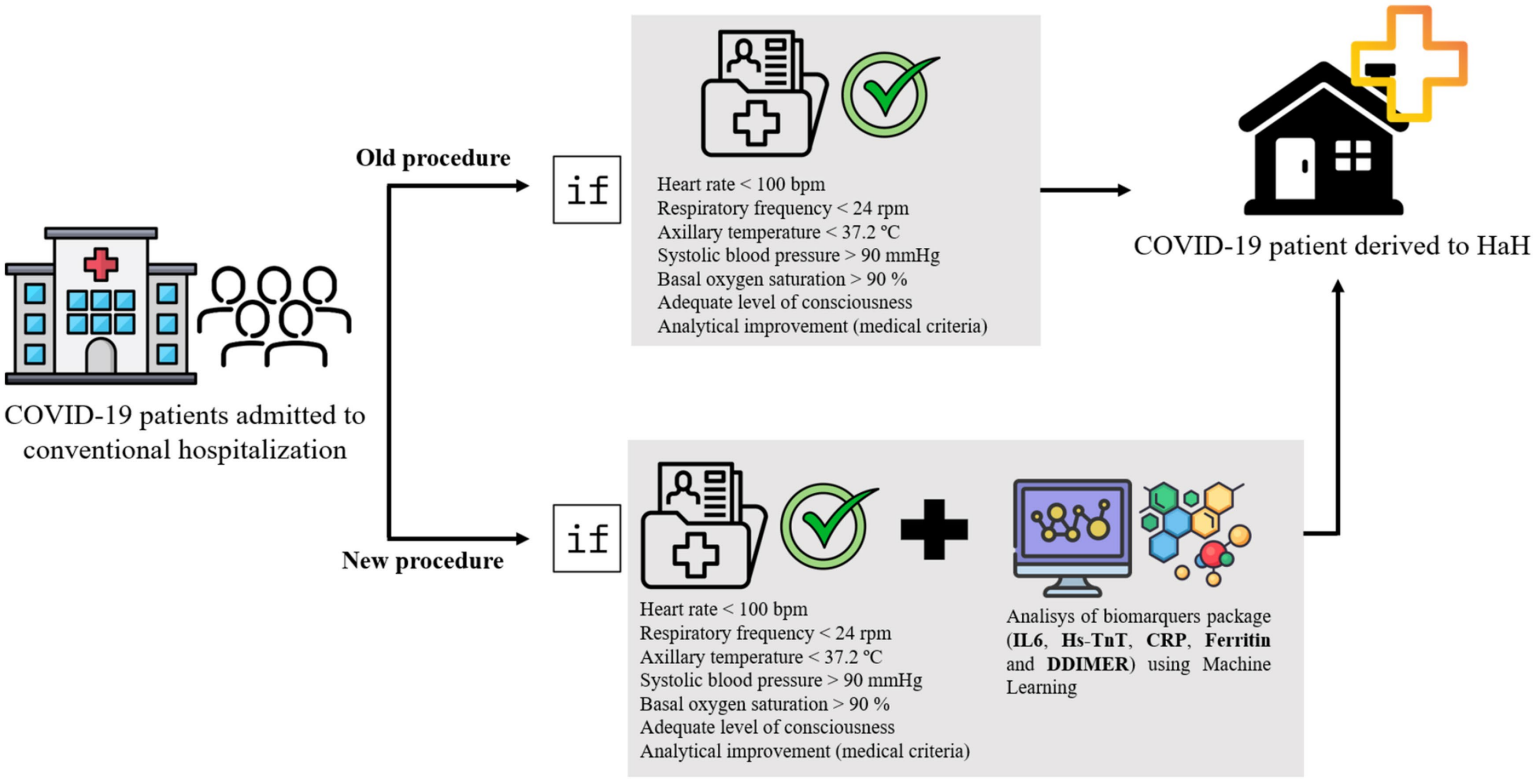
Diagram illustrating the integration of machine learning model outcomes into clinical decision-making.

During the COVID-19 pandemic, particularly during the initial three waves, hospitals experienced an influx of ICU patients that overwhelmed unprepared facilities. Emergency protocols were enacted to relieve this strain by facilitating patient recovery at home under the care of HaH services. However, these protocols, formulated during that time, lacked criteria based on biomarker values, despite numerous studies demonstrating the significance of Hs-TnT as a prognostic biomarker. This study illustrates that troponin levels were indicative of failure in the HaH process, necessitating patient readmission to conventional hospitalization. In addition, the application of ML algorithms can help clinicians make decisions regarding when to discharge patients from conventional hospitalization to HaH. A diagram illustrating the integration of machine learning model outcomes into clinical decision-making is presented in Figure 7.

## 7 Limitation

The results of this manuscript arise not from an experimental design but from data collected during the peak of the COVID-19 pandemic. Due to the effective implementation of emergency protocols, the resulting sample size is small, necessitating the use of a data augmentation algorithm, as discussed above. Nonetheless, these limitations do not diminish the significance of the results obtained.

## Data Availability

The raw data supporting the conclusions of this article will be made available by the authors without undue reservation.

## Footnotes

^1^ https://whahc-community.kenes.com/mod/page/view.php?id=1042
